# The evaluation of urban spatial quality and utility trade-offs for Post-COVID working preferences: a case study of Hong Kong

**DOI:** 10.1007/s44223-022-00020-x

**Published:** 2023-01-27

**Authors:** Qiwei Song, Zhiyi Dou, Waishan Qiu, Wenjing Li, Jingsong Wang, Jeroen van Ameijde, Dan Luo

**Affiliations:** 1 Arcadis IBI Group, Toronto, Canada; 2 School of Geographic Information Science, University of Queensland, St.Lucia, Australia; 3 Department of City and Regional Planning, Cornell University, Ithaca, USA; 4 Center for Spatial Information Science, The University of Tokyo, Tokyo, Japan; 5 School of Architecture, Tsinghua University, Beijing, China; 6 School of Architecture, The Chinese University of Hong Kong, Hong Kong SAR, China; 7 School of Architecture, University of Queensland, St.Lucia, Australia

**Keywords:** Urban data, Sustainable communities, Post-pandemic, Deep learning, Convolutional neural network, Trade-off

## Abstract

The formation of urban districts and the appeal of densely populated areas reflect a spatial equilibrium in which workers migrate to locations with greater urban vitality but diminished environmental qualities. However, the pandemic and associated health concerns have accelerated remote and hybrid work modes, altered people's sense of place and appreciation of urban density, and transformed perceptions of desirable places to live and work. This study presents a systematic method for evaluating the trade-offs between perceived urban environmental qualities and urban amenities by analysing post-pandemic urban residence preferences. By evaluating neighbourhood Street View Imagery (SVI) and urban amenity data, such as park sizes, the study collects subjective opinions from surveys on two working conditions (work-from-office or from-home). On this basis, several Machine Learning (ML) models were trained to predict the preference scores for both work modes. In light of the complexity of work-from-home preferences, the results demonstrate that the method predicts work-from-office scores with greater precision. In the post-pandemic era, the research aims to shed light on the development of a valuable instrument for driving and evaluating urban design strategies based on the potential self-organisation of work-life patterns and social profiles in designated neighbourhoods.

## Introduction

The purpose of contemporary cities is to provide people with convenient and healthy neighbourhood settings while promoting efficiency, resilience, and sustainability. However, the high densities of urban settlements with extensive commercial and industrial footprints have increased susceptibility to epidemic outbreaks and health concerns (Wang, 2021; X. Zhang et al., 2022). This has led to a rethinking of urban planning in the post-pandemic period (Bissell, 2021; Mehta, 2022a). In response to the pandemic, regulations encourage physical separation, and numerous industries now permit employees to work remotely permanently or in a hybrid capacity (M. Hu et al., 2021). When work patterns shift to remote work, urban mobility, commuting patterns, neighbourhood economies, housing prices, and street vitality are significantly affected (Florida et al., 2021; M. Hu et al., 2021; Mehta, 2022a).

People decide where to live based on a number of factors, including accessibility to urban amenities (Rode et al., 2017), access to public transportation (L. Yang et al., 2022), neighbourhood environment qualities (Y. Yang & Xiang, 2021), safety perceptions at the neighbourhood scale (Qiu et al., 2022; Song, Li, Li, et al., 2022; X. Xu et al., 2022), affordability of housing (Kang et al., 2021a, 2021b), and education quality of nearby schools (Wen et al., 2017). Consequently, in the post-pandemic context, given the changes in work mode, a significant mismatch has developed between the city's existing spatial form and function and the residents' life and work dynamics. When balancing desirable urban spatial qualities, the value of amenities, and employment opportunities, it has subtly but inexorably altered the housing preferences of individuals.

Given this changing environment, the working population is likely to face two distinct scenarios. On the one hand, people who primarily work from home may continue to reside in areas with shorter commute distances and travel times to offices, where this premium for convenience will no longer be relevant. However, they must still pay exorbitant rent, which is not desirable. Alternatively, because they only need to be physically present in the office once or twice per week, these employees may choose to commute longer distances from farther away (Florida et al., 2021). For these individuals, housing prices or rent in the suburbs are less expensive than in city centres, albeit with fewer amenities. This preference shift may be accelerated by the reduced demand for office space, resulting in vacancies and scepticism regarding the future desirability of office hubs, particularly in the expensive downtown areas of cities (Mehta, 2022a).

In the context of ensuring public health and well-being in response to urban disasters such as epidemics, the spatial distribution of urban resources exhibits both scarcity and abundance patterns (Wang, 2021). Therefore, urban design and construction at the neighbourhood scale will need to reconsider changing needs and functions, such as if a paradigm shift toward cultural and civic gathering activities necessitates more street spaces allocated for local neighbourhood uses (Florida et al., 2021; Mehta, 2022a; Wang, 2021). To comprehend these potential future policy changes and paradigm shifts, however, there is a lack of understanding of people's subjective housing selection preferences in relation to the complex and interwoven factors of urban context across multiple geospatial scales. The construction of models to predict neighbourhood development in the era of the "new normal" faces a number of significant challenges due to the following gaps:

- 1. Urban design characteristics have effects at multiple scales, and both the built and perceived environments are critical. When determining housing preferences, urban planning macro-level factors such as the density of places of interest (POI) and micro-level factors such as perceptions of the neighbourhood should be taken into account.
- 2. It is difficult to incorporate the subjective opinions of citizens regarding their pre- and post-pandemic residence preferences into an effective evaluation model that is also explicable. It is currently unknown whether and how the pandemic and related work-from-home scenario will affect housing preferences relative to the work-from-office condition.
- 3. To apply a model trained with existing variables collected from the current context to future scenarios, additional experiments are required.
- 4. People's actual actions and behaviours may differ from their stated preferences, and people's spontaneous activities may differ from their reported preferences.

Based on collected preference opinions from online questionnaires using Hong Kong as a case study site, this research aims to bridge the aforementioned knowledge gaps and supplement existing literature by integrating big data and the cutting-edge Computer Vision (CV) and Machine Learning (ML) models to provide an operable workflow for the topic. First, learnable weights are applied to urban characteristics from planning data at the macro-level and Street View Imagery (SVI) reflecting micro-level conditions in order to better comprehend the trade-offs in housing preference decisions. The second step is to collect intuitive subjective ratings from users using visual pairwise selection surveys based on both macro- and micro-level qualities of the neighbourhood to generate evaluation scores for each residence location in work-from-office and work-from-home conditions. Thirdly, we applied various ML and DL algorithms to investigate the effects of different built environment characteristics on residence preferences in both situations and to evaluate the performance of various models. Lastly, we validated the accuracy of stated preferences from surveys using massive amounts of data extracted from unplanned events such as real estate rent prices. This method can assess the accuracy with which subjective surveys reflect the preferences revealed and further validate the results. In conclusion, the research aims to contribute to the general understanding of how people's perceptions of housing preferences may change in response to different work modes and socioeconomic contexts when amenity and neighbourhood environment trade-offs are considered. As preliminary research on this topic, this study aims to provide meaningful inferences on evidence-based urban design strategies or guidelines for creating a more equitable and resilient future through urban transformation by gaining an understanding of subjective housing preferences. It has great future potential and applicability to predict neighbourhoods that are preferred in different work modes and to prioritise future redevelopment projects to meet the demand for poly-centric neighbourhoods.

## Literature review

### Residence preferences and utilities

Choosing a neighbourhood to live in is a complex process that requires a balance of various factors, such as proximity to essential amenities (Ferreira & Wong, 2020). In a competitive real estate market, housing prices reflect the revealed preferences of people's residential choices. Using housing price as an indicator of residential preference, there is a large body of literature employing various models such as the hedonic pricing model (Rosen, 1974). This model consists primarily of three attribute groups that are reported to influence the price, namely structural, location, and neighbourhood attributes (Huang et al., 2017). In addition to the structural attributes that capture the characteristics of the house's living condition, such as unit size and number of rooms, the location and neighbourhood attributes reflect the house's spatial locations within the city and other functions that residents find appealing (H. Li et al., 2019). Among these factors, public transportation facilities, such as subway stations and bus routes, facilitate access to employment and services. It is important to note that there is also a trade-off effect associated with housing preferences near public transportation, which is related to both convenience and noise and traffic intensity. For instance, research indicates that highway construction is less desired than bus and rail transit facilities (Agostini & Palmucci, 2008).

In addition, the access to service amenities has been studied in the past (Rode et al., 2017). These amenities include a variety of public service amenities, such as schools and hospitals, as well as private service facilities, such as shopping malls and restaurants, as well as cultural and recreational amenities, such as museums and sports facilities (Glaeser et al., 2018). Younger generations are attracted to the dense mating and friendship markets that these amenities can provide, which can help to retain population in these central urban areas (Florida et al., 2021). In addition to proximity to schools, the quality of the school education is related to the residential location for families with children, which is an important factor that may influence people's preferences (Wen et al., 2017).

Regarding public parks, it is widely recognised that they play an important role in the urban environment and provide numerous benefits for well-being, including recreational value and positive effects on physical and mental health (La Rosa, 2014). Recognized as an essential criterion for assessing the livability of a location, adequate access to green spaces can significantly influence people's residential decisions. Scholars discovered contradictory effects of green open spaces. On the one hand, in compact cities, proximity to urban parks is highly desirable (Netusil, 2013). However, in a North American city with much more sprawled urban fabric, no significant correlation can be found using Salt Lake City as case study site (H. Li et al., 2016).

### Spatial environmental qualities

According to numerous scholars, the success of the street environment is crucial to the development of a thriving urban centre (Jacobs, 1961; Y. Li et al., 2022; Montgomery, 1998). As a key category of public open space, the street not only serves the utilitarian function of transportation, but has also become more adaptable in terms of its programs, providing places for exchange and social interaction as well as venues for communities to reclaim for informal and formal activities, especially during lockdowns (Mehta, 2020). Important factors that can significantly influence people's physical activities (Lu, 2019), pedestrian route choice preferences (Sevtsuk et al., 2021), and the willingness to pay for housing or rent prices are neighbourhood street environment qualities (Qiu et al., 2022; J. Yang et al., 2021).

Previous research primarily relied on two metrics to assess the quality of built environment streets. Scholars have adopted objective measures employing remote sensing data and used GIS programs to spatially reflect the quality (Lee & Moudon, 2006). Typical objective characteristics of a dataset include building height, density, ratio of floor area to total area, park area sizes, number of specific facilities, access to public transportation, etc. (L. Yang et al., 2022). However, these macro-level planning-oriented variables may be less effective at capturing the daily human experience of site users in the micro-level street environment (Dubey et al., 2016). In contrast to objective factors, subjective measurement has been widely used in urban studies in the past, with interviews or surveys reflecting people's explicit opinions on the qualities of the spaces (Ewing et al., 2006; Lynch, 1960). However, this technique is limited to a small sample size and difficult to apply to a larger geographical area (H. Zhou et al., 2019). Consequently, with the availability of exploding urban big data such as SVI, recent urban studies have developed methods that use CV and ML algorithms to improve the efficacy of urban perceptions mapping on a scale never before seen (Gong et al., 2018; Qiu et al., 2023).

On the other hand, using CV semantic segmentation algorithms, researchers can extract the pixels of various street features from SVI as indices for objectively constructing perception scores (Zhao et al., 2017). For example, X. Li et al. (2015) created the revised green view index as a surrogate for the visual greenery, and the building and sky view factors were measured in a similar manner (Gong et al., 2018). Recently, researchers have attempted to quantify urban design perceptions (e.g., greenness, openness, enclosure, walkability, and imageability) utilising complex formulas derived from design theory and recombining the extracted view indices of key urban street features (Ma et al., 2021).

Using globally collected perception surveys such as Place Pulse Dataset 1.0 and 2.0, however, researchers could map psychological perceptual qualities such as safety, beauty, and boring (Dubey et al., 2016; Naik et al., 2014; Salesses et al., 2013). F. Zhang et al. (2018) predicted six perceptions in Beijing and Shanghai using extracted street characteristics as independent variables and perception survey results as training labels. Through the high-level extracted features, the study also revealed which streetscapes may result in varying perceptual qualities. The Place Pulse datasets were, however, collected globally, with limited results for China's streetscapes. To avoid potential bias, studies focusing on Chinese cities must rely on opinions gathered locally. Qiu et al. (2022), for instance, gathered expert opinions as training labels from a panel of experts. They subjectively measured five urban design-related perceptual qualities of Shanghai neighbourhoods using CV and ML models. They discovered that overall objective features outperform subjective perceptions in predicting housing prices, whereas subjective perceptions have a greater impact on individual objective features. Moreover, Song, Li, Li, et al. (2022) demonstrated that objective and subjective measurement of perceptions may exhibit contradictory spatial heterogeneity patterns even when mapping the same concept, indicating the need for future research to investigate the discrepancy and consistency of these two methods.

People remain cautious about social distancing in the post-pandemic era as Covid-19 waves return every few months, and scholars have identified a new set of dimensions for social activities in the public sphere due to hygiene concerns (Mehta, 2020). Changes in spatial distances between people, as well as shifts in work modes, have an impact on living preferences, travel, and commuting patterns. Taking into account the trade-off between accessibility to urban amenities and other assets, residents' preferences and perceptions of their neighbourhood environment may be subject to change. Utilizing SVI, prior research has focused on the micro-level characteristics and their effects on housing preferences using housing prices as a proxy (Kang et al., 2021a, 2021b; Kang et al., 2021a, 2021b; Qiu et al., 2022; J. Yang et al., 2021). Scholars have determined, for instance, that street-level greenery contributes to higher prices; in other words, people prefer neighbourhoods with greater exposure to vegetation (Fu et al., 2019). And through a place-based hedonic price model, Kang et al., (2021a, 2021b) determined that people prefer to reside in a neighbourhood with higher perceived safety and attractiveness. However, existing research focuses primarily on the pre-COVID era (L. Yang et al., 2022). It does not interpret how people's subjective residential preferences may have changed or which characteristics may influence residents' choices in the post-pandemic context (Florida et al., 2021).

## Data and methodology

In order to examine the effect of shifts in work patterns on housing preferences, our research focuses on the working population of design industries whose company offices are located in the city centre. Hong Kong is used as a case study example in response to contemporary issues.

**Fig. 1:**
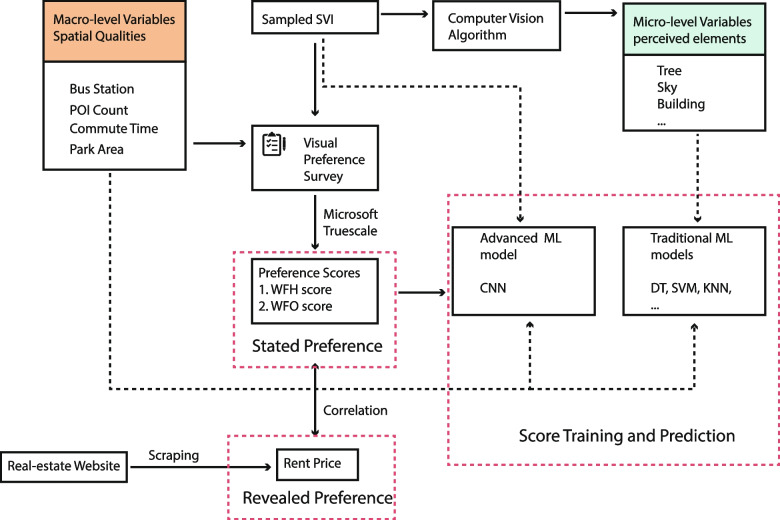
Method and workflow

We obtained the city's SVIs and the opinions of its residents through subjective rating of a randomly sampled SVI database. In this study, the innovative and crucial aspect of the workflow is that both macro-level planning-related objective spatial variables, such as points of interest, park size, commuting time to the city centre, and availability of public transportation, and micro-level perceived street quality are integrated and presented to the participants in order to consider all related factors under two working conditions comprehensively. The rating is based on data that has been altered to include revised neighbourhood conditions that simulate future development scenarios that are not representative of currently selected neighbourhoods. The variables from different scales reflect the complexity that real-world residents must consider when selecting a residence. Using a CV algorithm, the perceived street features are extracted from SVI data to reflect the physical neighbourhood environment. Various ML algorithms are utilised to evaluate the accuracy of the trained model of the work-from-home and work-in-office scores datasets. Last but not least, real estate rental data, which reveals human behaviour and housing preferences, is used to validate its correlations with stated preferences, i.e., subjectively evaluated scores (Fig. 1). This result will indicate how the two types of working scenarios may affect the housing preferences of residents, which will have significant implications for post-pandemic urban design strategies. It aims to provide a foundation for future research to predict human behaviour in response to changes in the sense of place and preferences for future development.

### Study area

The People's Republic of China's Hong Kong Special Administrative Region is a semi-autonomous administrative territory located in south of Guangdong Province, connecting to Shenzhen. Hong Kong Island, Kowloon, and the New Territories comprise its three geographical regions, and since 1999, the territory has been subdivided into 18 administrative districts. Approximately 7.5 million people inhabit the 1100 square kilometre land area, which is comprised of the historic urban core and a number of satellite towns. Due to its complex historical, political, and topographical conditions, the built land comprises only about one-fifth of the total area, and residents' living conditions are marked by high costs, compact apartments, and limited public open space (Kwok et al., 2021; Liu et al., 2020). Additionally, previous research has demonstrated that there are serious social inequality and segregation issues (Monkkonen & Zhang, 2011). In response to these alarming social challenges and in an effort to attract talent, the Hong Kong government has released the 2030 + strategic plan, which aims to improve the liveability in the compact, high-density city through a series of policies such as the provision of additional living units to increase capacity for future sustainable growth (Hong Kong Development Bureau & Hong Kong Planning Bureau, 2021). Residents can travel within one to two hours and live within walking distance of accessible open space in Hong Kong, which continues to have one of the highest public transportation usage rates among major developed cities. It enables professionals who live far away from their workplaces to commute via public transportation. However, as a result of the global pandemic, many companies have implemented the WFH policy for the first time in an effort to prevent the spread of infection, and the government has imposed social distancing mandates out of concern for public health (Wut et al., 2022). This disruption of working-living spatial dynamics and its consequential effect on the reconsideration of the trade-off between utilities and neighbourhood environment qualities has received less attention. Understanding people's subjective housing preferences in this new context can inspire future urban redevelopment projects and policy initiatives that contribute to a more sustainable and resilient metropolitan region, as envisioned by the Hong Kong 2030 + vision.

### Data collection and processing

**Fig. 2:**
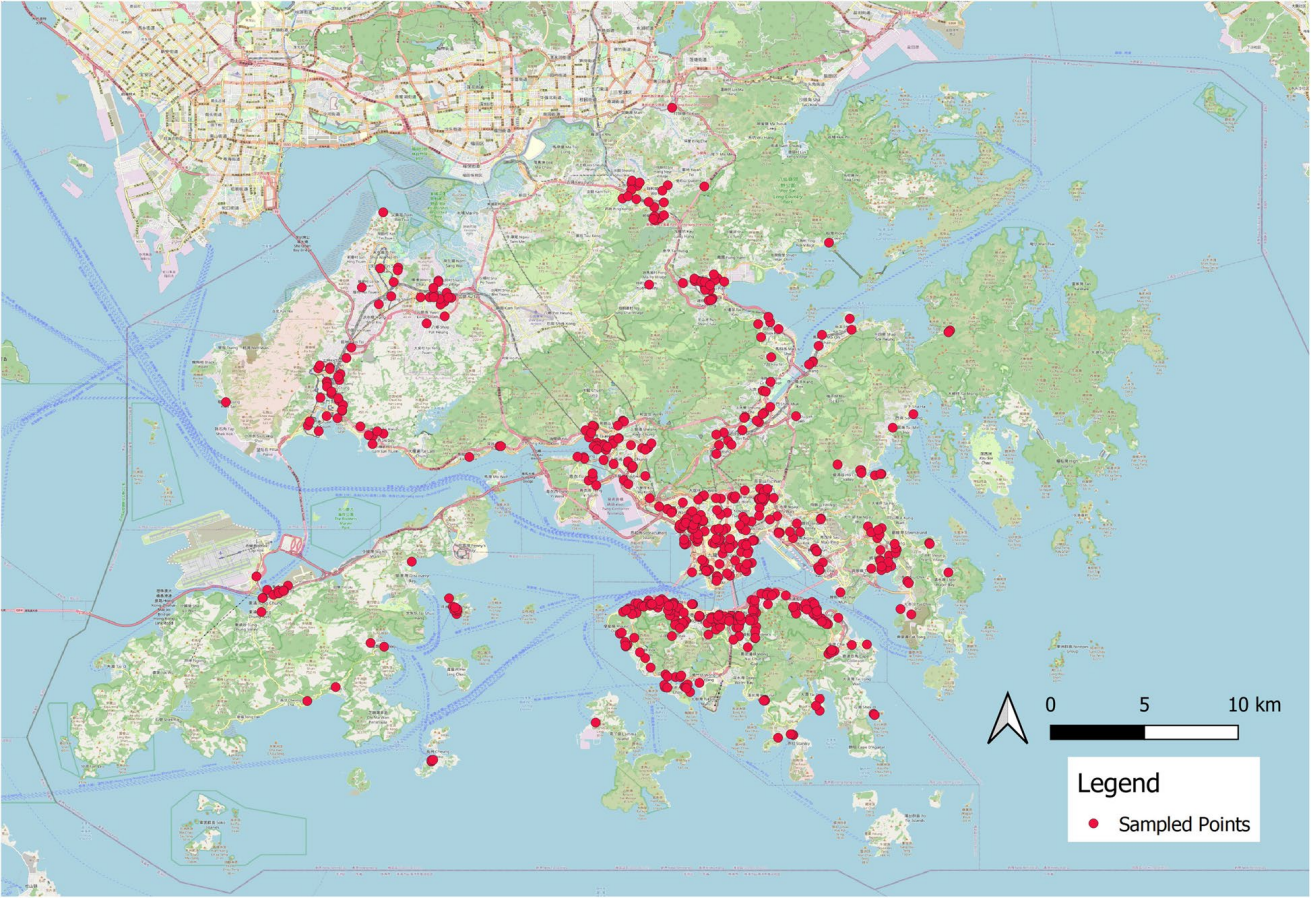
Five hundred seventy-eight sampled study points in Hong Kong

We obtained information about Hong Kong' s road network from Open Street Map, which provides free vector data. We sampled points along the road geometries at 50-m intervals using QGIS to create shapefiles (Qiu et al., 2022; H. Zhou et al., 2019). This resulted in the creation of 1,000 points in each district, and we accumulated 18,000 points throughout the city. To ensure that our training images cover the majority of urban area types and enhance the efficiency of the study, we randomly selected 578 points from the dataset (Fig.2). The sample size was determined by a compromise between the accuracy of ML prediction, the reliability of the survey, and the workload of the participants. According to Beleites et al. (2013), to obtain accurate ML prediction results, at least 100 samples or ten times the required number of independent variables must be collected. The traditional ML models used in our study extract street features using semantic segmentation; the CV algorithm extracted approximately 30 features from the scenes; therefore, our sample size must be at least 300 points; 578 points is nearly double the minimum requirement and is deemed adequate. Following previous urban perception mapping research (Qiu et al., 2023), we initially randomly sampled a similar number of points within each administrative district (approximately 32 points per district); however, due to the unique geography and settlement layout, the urban built land accounts for less than one-fifth of the total area, indicating that neighbourhoods can be concentrated across several districts. Therefore, based on the spatial layout, we manually balanced the number of sample points within the municipal districts to ensure that they could geographically represent the street environment of Hong Kong's urban and peri-urban neighbourhoods (Monkkonen & Zhang, 2011). We fed the geographic coordinates of these points into the Google Street View Static API and retrieved the platform's corresponding SVIs. Each scene is 800 by 400 pixels with a field of view (FOV) of 90 degrees and a pitch angle of 0. Despite the fact that some previous studies have utilised panorama views (Lu, 2019), these are susceptible to distortion, making it difficult for individuals to express their preferences. In order to maintain spatial and temporal continuity for subsequent visual survey comparison and machine learning (ML) process and to minimise potential biases, the 578 retrieved SVIs were cleaned to remove blurry, grey (blank), and highway and indoor images (Ito & Biljecki, 2021). This can help reflect the environment that residents will experience daily in the neighbourhood. And because our research site is Hong Kong, unlike previous studies conducted in northern climates such as Boston, where deciduous trees are the predominant street trees, the seasonality of street greenery is not a concern for our study (X. Li et al., 2015). Consequently, removal of winter images is not necessary. We manually replaced these invalid points with nearby points and then recalculated the SVI.

Significant criteria to measure street spatial quality that affect housing preferences are macro-level objective indicators (Song, Liu, et al., 2022a, 2022b, 2022c). In addition to the SVIs, we selected the 'number of POIs', 'number of bus stops', 'commute time to the city', and 'size of park area' as the key variables to represent the neighbourhood qualities. Hong Kong Geodata Store (https://geodata.gov.hk/gs/) is used to retrieve the spatial distribution of bus stations, POIs, and parks. And rent price information is crawled from the website of a real estate company, namely Centaline (https://hk.centanet.com/ info/index).

QGIS was used to process the macro-level datasets. Around the centre of each selected point, a buffer zone with a 1.5 km radius was created. We obtained the number of bus stops and points of interest, as well as park area sizes, within each point's buffer. The commute time was then determined based on the distance between the site and the city centre. These indicators are critical for enhancing the residents' livability and have been reported to influence housing prices (Huang et al., 2017; H. Li et al., 2019). The number of bus stops can be viewed as a proxy for the degree of mobility infrastructure, which provides crucial access to a residence and place of employment (Rode et al., 2017). The number of POIs reveals the urban spatial structure and provides data-driven guidance for regional spatial regulation and optimization. POI data documents diverse types of economic and social sectors and can be used to identify the grid units' functional zones and inform the human-scale vitality (Y. Hu & Han, 2019; Yue et al., 2017). The park sizes represent the overall environmental quality and contribute to subjective well-being and quality of life (M. Xu et al., 2017). Lastly, commute time highlights the reflection of travel behaviour of work-living experience as daily life; it is a crucial factor influencing residence experience on the long run and also becomes a safety concern in the post-COVID era (Florida et al., 2021).

**Fig. 3:**
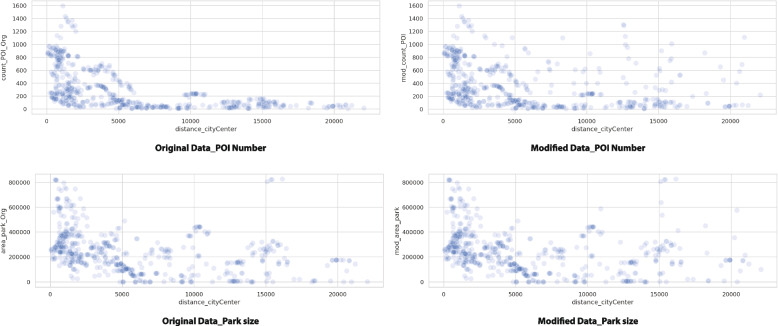
Study points scatter plot (original data VS modified data)

In order for the parameters of each variable to be more evenly distributed across the design parameter space, noise and variance were added to the existing data for a number of POIs and park area size (Fig. 3). This enables the data to include future possibilities that are currently absent.

### Perceived neighbourhood characteristics at micro-level

**Fig. 4:**
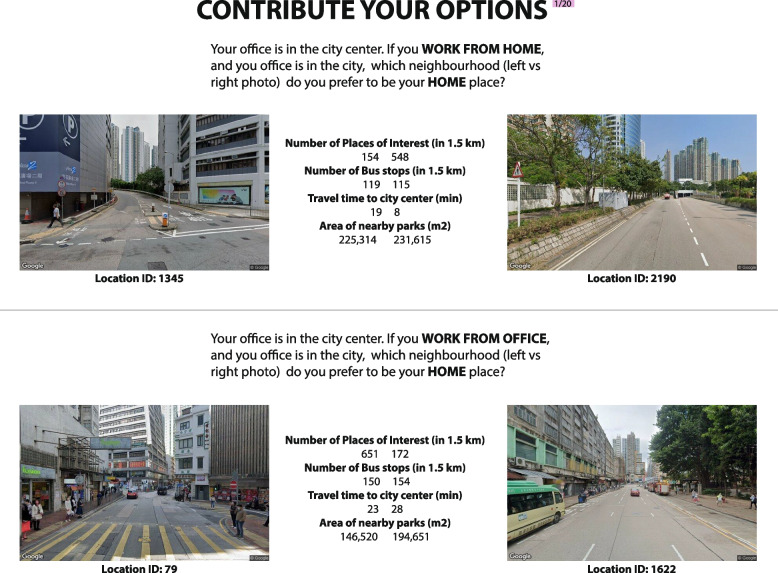
Questionnaire/Survey for Revealing Subjective Preference

We created an online visual questionnaire platform that solicits respondents' input on their preferences for living in two conditions (Fig. 4). On each page of the survey, participants are asked to compare pairwise SVIs and select their preferred scene by balancing the trade-off between perceived street environment quality based on a human vision of SVIs and macro-level urban spatial attributes such as commute time to the city centre. This illustrates the complicated logic behind how people choose their homes. On the basis of their working mode (work-from-home or in-office), respondents will assume that the office is located in the city centre and make a decision regarding their preferred neighbourhood (by clicking the preferred scene). Each page of the visual survey is divided into two parts; the top half of each page asks participants to evaluate the work-from-home condition, while the bottom half asks about the work-from-office condition. This means that each location will be evaluated under both the work-from-home and in-office criteria. It can help us compare how individuals evaluate living preferences when evaluating the same spatial qualities and utility access, but in different work scenarios.

Previous research suggests that pairwise comparisons improve the accuracy of personal preferences (Salesses et al., 2013). Using a dynamic comparison survey as opposed to a static score survey reduces personal bias, such as the tendency of some individuals to assign higher scores. The visual survey includes all 578 SVIs collected from Hong Kong. This sample size outperforms previous research, which also used a smaller training dataset; for example, Qiu et al. (2022) used 300 SVIs to predict urban design perceptions, and Ito and Biljecki (2021) collected 400 SVIs. The respondents were architectural students and young professionals between the ages of 20 and 35, and 3,718 valid responses were collected. Using Place Pulse Dataset 1.0, Salesses et al. (2013) demonstrated that people's preferences for street environment perceived qualities are not influenced by the demographic information of survey participants, such as age and gender, nor by the geographical locations of the respondents. Consequently, these responses can be generalised for future research focusing on various geographical locations. Nevertheless, future research can consider incorporating responses from other professions, which may provide varying answers due to the nature of work in various fields. The most frequently compared SVI in the working-from-home scenario achieved 31 comparisons, while the highest SVI in the working-from-office scenario achieved 27 comparisons. Each image was compared an average of 12.86 times, which exceeds the threshold of 12 comparisons suggested by Herbrich et al. (2006) for obtaining a reliable score estimate in the next step. It is close to the 16 times average comparisons in Salesses et al.(2013)'s study utilising Place Pulse Dataset 1.0 and significantly higher than Dubey et al. (2016)'s study utilising Place Pulse Dataset 2.0, which only achieved 3.4 times per image. Consequently, this dataset is deemed acceptable. The Microsoft True Skill algorithm, a Bayesian ranking method, was utilised to faithfully convert survey preferences into scores based on a two-player game mechanism (Minka et al., 2018).

### Extracting micro-level perceived neighbourhood characteristics

**Fig. 5:**
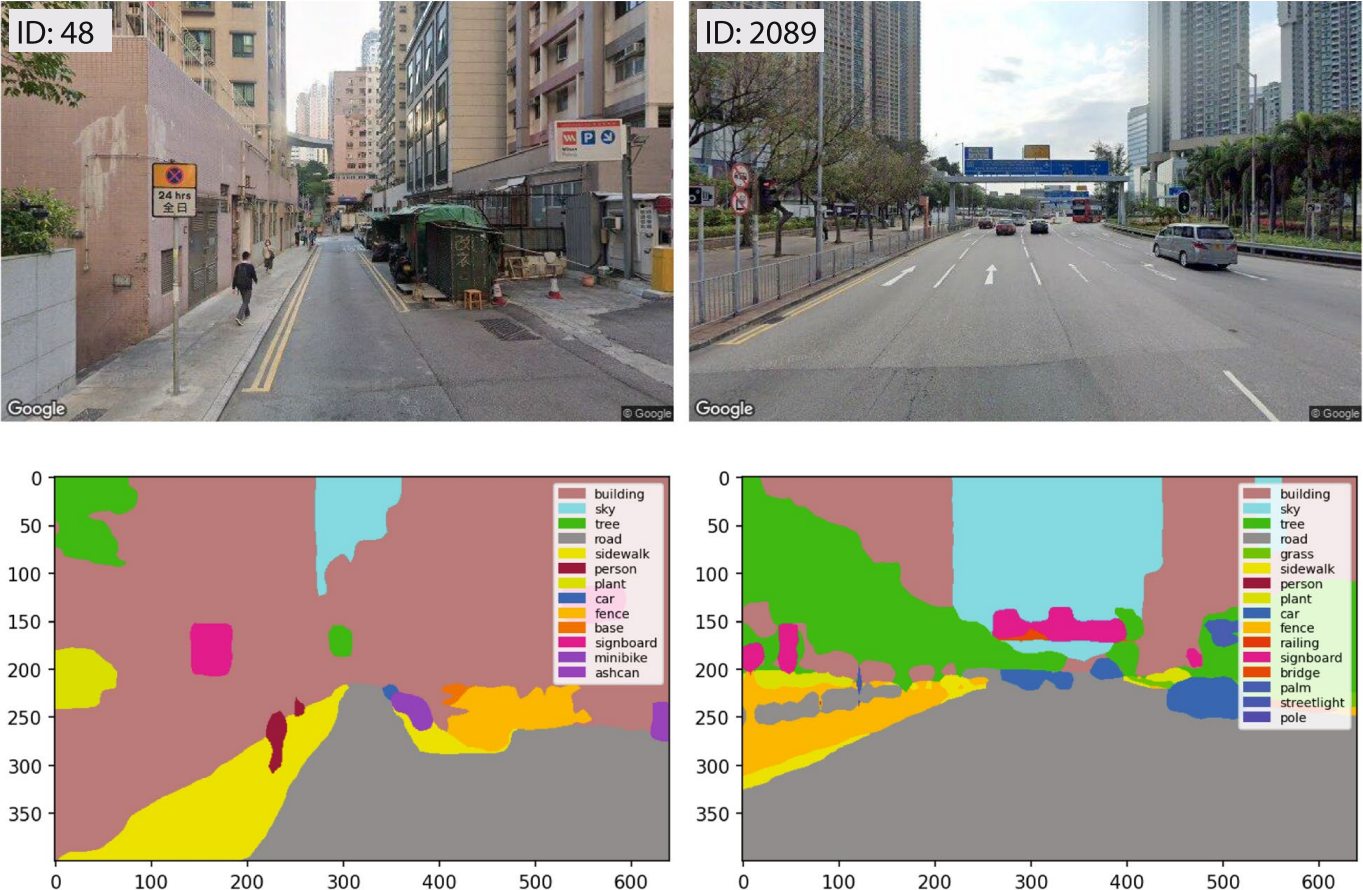
SVI inputs and PSPNet semantic segmentation outputs

SVI physical characteristics can serve as proxies for perceived street characteristics (Ewing & Handy, 2009). Prior research has quantified their numbers in order to further establish a connection with ML models for prediction (Qiu et al., 2022; Song, Li, Li, et al., 2022). We used the pre-trained Pyramid Scene Parsing Network (PSPNet) algorithm (Fig. 5) with the ADE20K dataset to efficiently segment the physical features from the SVI scenes and compute the pixel ratio of each type of street element (Zhao et al., 2017; B. Zhou et al., 2018). The CV algorithm achieved a pixel-level precision of 93.4% and has been extensively utilised in previous urban studies (Qiu et al., 2023; Song, Li, Qiu, et al., 2022). More than 30 types of street features were extracted from the SVIs following the procedure. We chose eight major elements (i.e., building, sky, road, signboard, wall, tree, sidewalk, and fence) for the next step of ML model training and prediction purposes, after removing ephemeral objects such as automobiles and those with little significance in the scenes.

### ML, DL Model training and validation

Following Qiu et al. (2021)'s approach to using traditional ML algorithms for prediction, the dependent variables of this study's intended output are two types of preference scores (work-from-home and work-from-office). Independent variables consist of the extracted pixel ratios of selected eight features and objective spatial qualities. The nine ML models utilised in this study are Linear Regression and Random Forest (RF), etc.

To select the optimal ML model, we evaluated the performance of various ML algorithms using the Root Mean Square Error (RMSE) as the loss function, which has advantages in punishing significant errors. The lower the RMSE value, the more accurate a prediction model. In addition, we calculated the R-Squared (R2) value, which effectively explains the model fit by indicating how much of the variance of a dependent variable can be explained by independent variables in a regression model. The model results are shown in Table 1.

**Table 1:** Performance of traditional ML algorithms

Model	**Q1_Home**		**Q2_Office**	
	R2	RMSE	R2	RMSE
**Random Forest (RF)**	0.11	0.18	0.23^a^	0.19^a^
**Decision Tree (DT)**	0.14^a^	0.18^a^	0.21	0.19
**Voting Selection (VS)**	0.08	0.18	0.23^a^	0.19^a^
**Gaussian (GS)**	0.08	0.18	0.23^a^	0.19^a^
**ADA Boost (ADAB)**	0.04	0.18	0.22	0.19
**K-Nearest Neighbours (KNN)**	-0.11	0.2	0.06	0.21
**Support Vector Machine (SVM)**	0.06	0.18	0.14	0.2
**Ordinary Least Square (OLS)**	0.08	0.18	0.23^a^	0.19^a^
**Bagging Regression (BR)**	0.07	0.18	0.18	0.19

^a ^represents the best-performing model

The best traditional machine learning method for predicting the Q1_home score is DT, which achieved an RMSE of 0.18 and an R2 of 0.14. Four ML models had identical performance for the Q2 Office score, with RMSE of 0.19 and R2 of 0.23; the R2 of these models explained more variances than the DT for Q1 scores. However, their results are regarded as having a low degree of predictive accuracy because their criteria fall below typical threshold values (Qiu et al., 2022).

Traditional machine learning methods rely heavily on training features at the highest level. Although applying semantic segmentation to pre-process street scenes provides information on quantities of each type of feature and makes it interpretable (Song, Li, Qiu, et al., 2022), it is less efficient to translate the overall ambience, style, and textures as well as the details of the streetscape; the information tends to be lost after extraction. Therefore, this study has advanced towards Deep Learning (DL) by employing a custom Convolutional Neural Network (CNN) to train the dataset (F. Zhang et al., 2018).

**Fig. 6:**
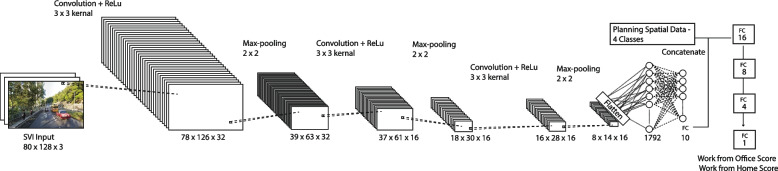
Customised CNN model architecture

By repeatedly stacking a 3 × 3 convolution kernel and a 2 × 2 max pooling layer three times, the dropout function is employed for regularisation to prevent overfitting issues. Convolutional layers culminate in layers with complete connectivity. In order to use both planning spatial quality variables and CNN output data readings from SVI as independent variables, the model concentrates them and constructs densely interconnected layers with the final output of a score (Fig. 6). Both scores have significantly improved compared to the results of conventional ML models (Table 2). The RMSE for the Q1_Home score was 0.0195, and R2 was 0.34. Although the R2 of the Work-from-Home scenario does not demonstrate a significant correlation, CNN achieved much greater accuracy for the Q2_Office scenario, which had an RMSE of 0.0045 and an R2 of 0.779. The R2 value of Q2_Office outperforms previous studies that used SVI to predict subjective perception scores, whose R2 values range from 0.47 to 0.61 (Qiu et al., 2022; Song, Li, Qiu, et al., 2022). Due to the ratio between the training and testing datasets, the model may be susceptible to an overfitting issue. The results indicate that for the Work-from-Office score, CNN has proven to be applicable for predicting preference scores based on street scenes and neighbourhood spatial quality factors.

**Table 2:** Performance of CNN Model

	**Q1_Home**		**Q2_Office**	
**CNN Model**	R2	RMSE	R2	RMSE
**Performance**	0.34	0.0195	0.779	0.0045

### Correlation results between stated preferences and revealed preferences

**Fig. 7:**
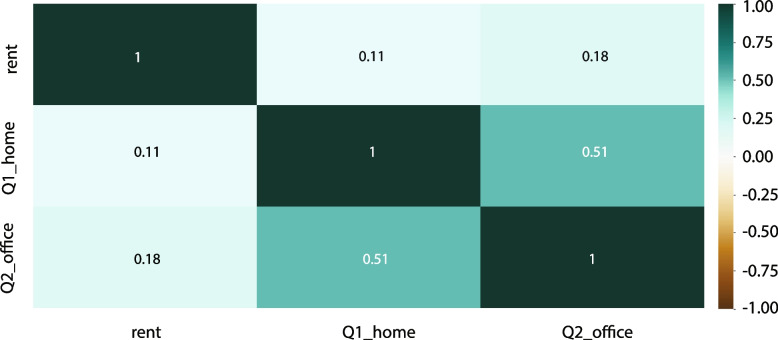
Pearson correlation between scores and rent prices

The stated preferences of work-from-home and work-from-office scores are compared to the revealed preferences, which are the rent prices collected from a Hong Kong real estate website. The results (Fig. 7) demonstrated that the work-from-home score (Q1_home) and the work-from-office score (Q2_office) are highly positively correlated (coefficient = 0.51). Although the correlation between Q1, Q2 and rent price is less than 0.2, which indicates a weak correlation, the coefficient between work from office score and rent is approximately 64% higher than the coefficient between work from home score and rent price.

## Discussion

### Interpretations of CNN Model

In general, the CNN model is more accurate than traditional ML models at predicting both types of scores based on the neighbourhood environment and urban amenities trade-off, and it captures the nuances of residents' opinions considerably better. It is reported, however, that the model work-from-office score has a greater R2, which explains more score variance, and a lower RMSE, which indicates less error in predicting scores. This indicates that our CNN model explains the work-from-office scenario better than the work-from-home circumstance. Our validation using rent price as the revealed preference demonstrates further that the work-from-home score correlates less with rent. This result demonstrates conclusively that people's housing preferences have changed significantly in relation to their work mode.

Multiple hypotheses can explain these results. Firstly, housing preferences are jointly influenced by a large number of factors; the spatial qualities captured by our model could be expanded, particularly for the work-from-home scenario. Also, people may have different needs at various stages of their lives; for instance, young adults may want to work in the city, enjoy more urban amenities, and socialise more frequently. While forming a family with children, work-from-home parents may take into account the educational quality of nearby schools, the availability of day care facilities, the flexibility of their own jobs, and other factors. Secondly, due to the disruption of mobility patterns and the shift in work modes, depending on job types, certain factors may have a greater influence on choices. Access to a variety of amenities no longer confers a premium on convenience for people who work remotely. Due to health concerns, previous research in China revealed that the implicit price of metro access around Chengdu decreased for a time. This indicates that the value of convenient access to public transportation amenities is decreasing due to shift in work modes. Nevertheless, based on the prediction model, the price is likely to rebound in the long term (L. Yang et al., 2022), and this requires further research. Thirdly, commuting time may be the most important factor in deciding where to live for people who need to commute to the office, as those who use public transportation more frequently are more susceptible to epidemic diseases (X. Zhang et al., 2022). In the current post-pandemic context, commuting time may be the dominant factor that trumps all other indicators for reducing health-related risks. Depending on the office location, people may choose to continue living in urban areas if their homes do not provide sufficient space for a functioning office (Wut et al., 2022). Fourthly, regarding access to amenities, a poll conducted during the first pandemic wave revealed that 40% of city dwellers considered moving to the suburbs to enjoy more private amenities, and they may never return (Hart, 2020). How people view this in the long run is unclear and requires further research. Fifthly, the cessation of mobility and transition to a hybrid work mode facilitates a shift in the sense of place and how people perceive their neighbourhood environment (Bissell, 2021). During the lockdown, people must rely more on their neighbours, and after the pandemic, these interactions have fostered a previously absent sense of social connectedness (Ottoni et al., 2022). In addition, physical separation has spawned new proxemics (Mehta, 2020), which foster new dimensions for social space. In conclusion, urban parks are increasingly recognised for their unique benefits to mental and physical health. Researchers discovered that greater access to parks in neighbourhoods with higher population densities can encourage young people to remain physically active through exercise (Mitra et al., 2020). People may prefer communities with more open space, wider sidewalks, and ample bike lanes. Nonetheless, it is important to note that these dynamic changes are more likely to create new opportunities in neighbourhoods with a medium-to-low population density, whereas this may not be the case in dense neighbourhoods. These observations raise concerns about social inequality, which may be exacerbated by the pandemic (Florida et al., 2021; Y. Yang & Xiang, 2021). The Hong Kong 2030 + Vision Plan has envisioned adding more green space; where and how to implement them through projects, taking into account the various work modes, are worthy of future research.

### Future urban spatial patterns

FLORIDA et al. (2021) have predicted the effects of the global pandemic and the potential future of the world post-pandemic. They predict that although cities will rebound in the long run and on a large scale, there may be temporary or permanent morphological changes on a smaller scale. Taking into account the urban amenity trade-off and the neighbourhood environment, various groups of people may have contradictory housing preferences. Some people may choose to live in suburban areas, and we anticipate that their 'urban' characteristics will increase and their functional mix will evolve. Due to the reasons stated in the previous section, other groups of people may desire to live in urban centres and enjoy the convenience of accessing amenities without the need for public transportation. This may indicate a more polycentric pattern of urbanisation that links diverse types of neighbourhoods or communities.

In Hong Kong, the WFH policy, despite being an indispensable component in addressing health concerns, is not devoid of pitfalls. The lack of resources makes it difficult for certain sociodemographic groups of Hong Kong residents to adopt this policy on a long-term basis, in contrast to cities in North America, where residents typically live in much larger homes that allow for the allocation of space for work. Individuals who are able to achieve greater WFH effectiveness are more likely to prefer this hybrid mode and more willing to continue after the pandemic, according to studies (Wong et al., 2020). Finally, scholars argued that these types of neighbourhood choices are ideal in the real world because residents may have limited experience with residential choices and rely on a limited social network for information. Such individual imperfection results in a certain bias in the estimation of neighbourhood preferences; therefore, this imperfection factor must also be accounted for in the housing preference prediction model (Ferreira & Wong, 2020).

### Implications on policies

The research findings on the shift in housing preferences have the potential to be applied in other regions of the world, especially in comparable Asian metropolitan cities with a high population density, such as Tokyo and Singapore. This may also be appropriate for compact and transit-oriented development. However, if the urban morphologies are located in a low-density context with a starkly different urban–rural spatial pattern that is primarily car-oriented, it may have minimal effects due to the lifestyle differences. For instance, scholars have suggested that people in these neighbourhoods would rely on different means to access open space, such as private automobiles, and that a different strategy would be required to expand the public space around these neighbourhoods compared to urban centres (H. Li et al., 2016; Mehta, 2022b). Furthermore, scholars hypothesised that occupations with a higher proportion of knowledge-based work, such as some tech-related industry jobs, are likely to continue this work-from-home trend (Florida et al., 2021). Consequently, unlike Hong Kong, this shift in housing preferences may have already occurred in certain cities, such as San Francisco, which are home to the major campuses of these technology companies.

These observations and findings could inform changes in guidelines to reimagine urban morphology, open spaces, and public gathering places, ultimately contributing to the objective of creating a healthy city. According to research, elements of the built environment, such as urban block morphologies, road networks, building configurations, and socioeconomic factors, influence COVID incidence and mortality rates (M. Hu et al., 2021; Kwok et al., 2021). Comparing the Compact City principles and pedestrian-oriented development to other forms of sustainable urbanism with a moderate population density, the pandemic has raised many questions about which urban planning model ought to be promoted. There is no conclusive evidence linking population density and the pandemic, as studies have yielded conflicting results. According to studies, the decline of COVID is more closely related to mobility patterns than to building density, as people who can access neighbourhood retail stores without having to travel far are safer (Y. Yang & Xiang, 2021). These findings further substantiate the advantages of well-planned compact developments in the direction of a self-sustaining model (X. Zhang et al., 2022). On the other hand, they suggest the need for potential changes to land-use planning. The pandemic demonstrates the fragility of retail main streets and emphasises the importance of neighbourhood shops for fulfilling daily shopping needs. The transformation of shopping centres to incorporate functions more suited to local needs and work-from-home professionals contributes to the development of a neighbourhood ecosystem and commons. There is an opportunity to create a new image of a social and civic neighbourhood by adding more layers of urban vibrancy and activities (Florida et al., 2021; Mehta, 2022a). The pandemic necessitates adjustments to the city's current planning and design methods. We should learn from these ongoing lessons, construct future cities that are healthier and more resilient (Lak et al., 2021), and be more prepared for future uncertainties (Wang, 2021).

## Limitations

This study is a proof-of-concept examination of a methodology and workflow that has limitations. First, we collected data from design students and young professionals to analyse the preferences of Hong Kong's working population. In other words, our study focuses primarily on the working preferences of industry professions related to architectural design. To expand our knowledge, we intend to solicit the opinions of workers or professionals from other fields for future projects. Due to the varied characteristics of the work, it may reveal diverse subjective preferences. To ensure the validity of the sampling data, it is advantageous to classify and select various types of home and office populations based on the objectives of future studies. Second, we plan to use more scientific sampling methods in our future research. For example, the classification of case study sites can be based on macroscopic a priori data such as scale and accessibility to amenities as evaluation indicators, and then random sampling and subjective evaluation of similar spaces in different types of spaces. And to complement existing quantitative frameworks, a mixed research method can provide additional value to reveal the dynamic correlation between spatial form and behavioural space. Third, the use of SVI images can be expanded by, for instance, providing participants with a series of different neighbourhood images to compare and gain a better intuitive understanding of perceived neighbourhood qualities. Fourth, the survey can collect the opinions of more respondents, resulting in more accurate subjective scores. Fifth, ML models can be improved, as PSPNet' s parsing of SVI images and use of ML results in inaccurate predictions. In our case, however, using CNN and realistic urban scenes reduces the error rate. In addition to spatial objective quality variables, the prediction model may also incorporate high- and low-level features and street images in order to predict preference scores (Song, Li, Qiu, et al., 2022). Previous research has demonstrated that subjective perception is superior to objective characteristics for predicting housing prices. Thus, the function of urban design features can be investigated further (Qiu et al., 2022). Sixth, more objective factors can be added to reflect the extent to which particular residents make well-informed decisions. Collecting pre- and post-pandemic rent or housing prices may serve as a validation dataset for comparing with subjective opinions (L. Yang et al., 2022). In addition to these limitations and opportunities for future development, the methodology framework presented in this paper has been demonstrated to perform statistically and accurately. It can be applied to other cities while conducting a systematic survey involving a wide variety of groups and neighbourhood samples.

## Conclusions

This study fills in gaps in the understanding of urban residents' residence preferences by examining the trade-offs between urban spatial qualities and public facilities for various work modes. It has utilised numerous ML and DL models to predict reference scores for various scenarios (work-from-home VS work-from-office). The research is based on 578 sampled study areas from Hong Kong and uses SVI to quantify human neighbourhood environment perceptions via pixel ratios in traditional ML models or DL using a custom CNN model. It has objectively measured urban spatial quality indicators and combined these with perceived scenes to elicit people's real subjective opinions through a survey gathering the preferences of the working population on different work modes.

Our evaluation method innovatively integrates street scenes, objective neighbourhood data, and subjective opinions into a DL prediction model that can comprehensively predict and inform neighbourhood streetscape transformations to be better oriented towards a post-pandemic condition when selecting different work modes. As long as SVI datasets and spatial quality data are accessible, the method can be applied to a variety of global locations. It provides a workflow for examining housing preferences for other work population groups and new research questions; for example, the objective attributes can include specific data, such as crime rates or rent prices, to expand the independent variables. This research aims to one day enable the evaluation of neighbourhoods based on the preferences of a variety of working population groups by predicting their competitiveness in attracting target residents by toggling on and off selected work modes, thereby developing strategic economic sectors and preparing development plans.

## Data Availability

The datasets used and analysed during the current study are available from the corresponding author upon reasonable request.
